# The Cholera

**Published:** 1910-09-24

**Authors:** 


// The Hospital

A JOURNAL OF

The Medical Sciences and Hospital Administration.

NEW SERIES. No. 189, VOL. VII. [No. 1261, VOL. XLVIII.] SATURDAY, SEPTEMBER 24, 1910.

### THE CHOLERA.

THE news that the cholera is making headway in Italy, in spite of the strenuous efforts of the Roman health authorities to keep the disease within bounds, makes it imperative for us to consider in how far we, in these islands, are guarded against an epidemic outbreak of the plague. Our attitude with regard to epidemic disease is somewhat different from what it used to be several decades ago. Then the mere mention of the approach of such a foe was sufficient to create a scare. Nowadays we have learned to look lightly on such things, secure in our knowledge that we have a competent sanitary authority to look after the interests of the community, and contemptuously indifferent, with the indifference to danger that familiarity—and more especially a false familiarity—breeds. Certain air-borne epidemic diseases are constantly with us, and mild waves of other complaints occur from time to time, so that the public has many opportunities for seeing how easy it is in modern times to stem the tide of an epidemic provided proper precautions have been taken. A case of small-pox hardly excites sufficient interest nowadays to serve as a subject for discussion, and a sporadic outbreak of typhus, the once so much dreaded gaol fever which is still rampant in some quarters on the Continent, is only deemed worthy a small paragraph in minor type in the daily newspapers. Gradually we are becoming more alive to the value of sanitary prophylaxis in dealing with these disorders, and it is scarcely a matter for wonder that the public should have put its faith so firmly in the capability of the health authorities to deal promptly and effectually with any case of cholera that may be notified in this country. While that faith is admirable enough, and a gratifying tribute to the endeavours of sanitarians, it is worth while to question whether it is, in our present circumstances and in view of the grave advance which the disease is making on the Continent, entirely justified.

To a casual observer it may seem that we are at the present moment sufficiently well protected against the disease. In all our great cities adequate sanitary regulations exist and are enforced. Our water supplies are nearly everywhere above suspicion, and the health authorities are fully awake to the need of thoroughly guarding against the dangers that lurk in dirt and insanitation. Above all, it may be argued that the public at large is sufficiently educated in health matters not to neglect any of the ordinary precautions that would have to be taken in the event of sporadic cases of cholera being notified in London or elsewhere. So, in the circumstances, it would appear that we are efficiently on the defensive, and that there is no reason to anticipate an outbreak on anything like the same scale as in Italy or Russia, or a mortality at all commensurate with that recorded in these countries.

But it is well to look for a moment at the other side of the picture and to reflect that however much we have advanced in matters concerning public hygiene there yet remains a vast amount of work to be done before our large cities—and small towns too for that matter—can be said to be as adequately protected from the disease as they ought to be. Anyone who is acquainted with the poorer back streets of London, especially those that radiate from the river centres—exactly the points where the danger is to be anticipated—will realise that strict attention to sanitation and a rigid enforcement of existing regulations are absolutely necessary to safeguard the community should the disease actually reach our shores. We have sufficiently learned the futility of attempting prophylactic measures once the disease has actually established itself—the history of cholera in Russia during the last two years is a convincing proof of this fact—and that to be effective such measures need to be attempted long before there is anything like a "scare." It is unfortunate that the public needs startling lessons to drive home the argument in favour of prevention in cases of epidemic disease of such virulence as cholera, and we would venture to suggest that the authorities might well take time by the forelock and prepare for emergencies by educating the masses so far as this special disease is concerned. This is a matter in which the lay press may render vigorous support by explaining to the public the main facts concerning the disease, its incidence, its bacteriology, and its course. No great technical knowledge is required to elucidate the matter, and the health authorities, we feel sure, will welcome any assistance given them in this direction. A series of leaflets, stating in clear and simple language what precautions are necessary, what dangers are to be anticipated, and in what manner these dangers may be circumvented, will prove of distinct value.

There is no reason whatever to develop a scare or to frighten the public needlessly, but there is every reason to explain that we are not so adequately protected as we might be, and to adopt the most careful and prepared tactics in dealing with the threatening danger. We are assured on all sides that there is no danger, and we are willing to believe that the chances of a cholera-infected immigrant landing at a London docks are remote, seeing that we are protected by our insularity and are not exposed to the danger of a widely extended land frontier as is the case with Holland and Italy. But it is worth while bearing in mind that there is an old rule that states that cholera comes from the sea, and it is strikingly significant that most of the Dutch cases last year were notified in seaport towns. If a further reminder of the maritime risks were required it is to be found in the history of the more recent Italian and Dalmatian cases, all of which were also notified from seaport towns. In the circumstances it is as well to take the utmost precautionary measures, and none of these measures can be really efficient or trustworthy unless the public is fully alive to the danger that lies in insanitation and dirt.

